# Genetic diversity amongst isolates of *Neospora caninum*, and the development of a multiplex assay for the detection of distinct strains

**DOI:** 10.1016/j.mcp.2009.01.006

**Published:** 2009-06

**Authors:** S. Al-Qassab, M.P. Reichel, A. Ivens, J.T. Ellis

**Affiliations:** a Department of Medical and Molecular Biosciences, University of Technology, Sydney, P.O. Box 123, Broadway, New South Wales 2007, Australia; b Wellcome Trust Sanger Centre, Hinxton Hall, Hinxton, Cambs, UK

**Keywords:** *Neospora caninum*, Cattle, PCR, Genotyping, Multiplex, Repetitive DNA

## Abstract

Infection with *Neospora caninum* is regarded as a significant cause of abortion in cattle. Despite the economic impact of this infection, relatively little is known about the biology of this parasite. In this study, mini and microsatellite DNAs were detected in the genome of *N. caninum* and eight loci were identified that each contained repetitive DNA which was polymorphic among different isolates of this parasite. A multiplex PCR assay was developed for the detection of genetic variation within *N. caninum* based on length polymorphism associated with three different repetitive markers. The utility of the multiplex PCR was demonstrated in that it was able to distinguish amongst strains of *N. caninum* used as either vaccine or challenge strains in animal vaccination experiments and that it could genotype *N. caninum* associated with naturally acquired infections of animals. The multiplex PCR is simple, rapid, informative and sensitive and should provide a valuable tool for further studies on the epidemiology of *N. caninum* in different host species.

## 1 Introduction

*Neospora caninum* is a cyst-forming apicomplexan parasite, which can invade many different cell types and tissues. It causes stillbirth and abortion in cattle and neuromuscular disorders in dogs, although infections have been reported in several other host species [1–4]. Neosporosis is now considered to be the main cause of infectious bovine abortion around the world [3,5,6].

There are marked differences in pathogenicity (virulence) and growth rates amongst isolates of *N. caninum* [7–9]. For example, NC-Liverpool causes a substantial inflammatory response in the central nervous system of the mouse, leading to disease, whereas NC-Nowra does not [10]. Therefore, the biological and genetic diversity among *N. caninum* isolates could impact on the pathogenesis of neosporosis in both cattle and dogs and might also be an important consideration in the design and application of vaccines against neosporosis.

Diversity in biological characteristics may also have considerable implications for our understanding of the epidemiology of neosporosis. Detailed information about the genetic diversity among different geographical isolates of *N. caninum*, however, is scant. Genetic diversity among isolates of *N. caninum* has been detected using ribosomal DNA sequencing; the analysis of inverted repetitive DNA and microsatellite repetitive DNA [7,8,11,12]. To understand genetic diversity within *N. caninum* and the contribution of these genetic differences to the heterogeneity in disease manifestation as well as transmission patterns for developing new strategies for vaccination or diagnosis, the detection of genetic variation (“strain typing”) using suitable markers is essential. Any such markers should be simple, stable, rapid, reproducible and discriminatory [13,14].

Mini and microsatellites are repetitive DNA sequences in the genomes of eukaryotic organisms, containing tandem repeats of a DNA motif. They are highly polymorphic in sequence and length [15,16]. Microsatellites are short, tandem repeats (2–6 bases long), whereas minisatellites have longer repeat units (8–100) [17,18].

Recently, a study of microsatellite markers described genetic diversity amongst nine isolates of *N. caninum* [12], and a number of polymorphisms were detected. No relationship was found between the organisation of the nucleotide repeat and the degree of polymorphism. Multilocus analysis revealed that each of the nine isolates displayed a unique profile and revealed no association between the DNA profile and host or geographical origin.

The aim of the present study was to extend such studies to investigate other repetitive DNA found in the genome of *N. caninum*. Specifically we focused on more complex repeats, such as minisatellites, to determine the genetic diversity among distinct isolates. In addition we developed a multiplex PCR which can be used for the characterization and differentiation of isolates of *N. caninum*.

## 2 Materials and methods

### 2.1 Isolates of *N. caninum*

The isolates of *N. caninum* used in this study are listed in Table 1. Nine of them are currently maintained in culture at the University of Technology Sydney (NC1, NC-Liverpool, NC-Nowra, NC-SweB1, BPA1, BPA6, NC-Beef, NC-Illinois and JAP1) using standard procedures described elsewhere [10,19]. Genomic DNA from 16 additional isolates were kindly provided by colleagues (NC-GER1, 2, 3, 4, 5, 6, 8 and NC-GER9 by Dr G. Schares (Germany), NC-LivB1 and NC-LivB2 by Dr D. Williams (UK), NC-Bahia by Dr L. Gondim (Brazil), WA-K9 by Dr L. McInnes (Australia), NcNZ1, NcNZ2, NcNZ3 by Laryssa Howe (New Zealand) and NcIs491 by Dr V. Shkap (Israel)). Twelve isolates originated from dogs and 13 were from cattle.

Tachyzoites were maintained in African Green Monkey Kidney (Vero) cells seeded in 75 cm^2^ vented flasks. Cells and parasites were grown in RPMI-1640 medium (Sigma) supplemented with 50 U/ml of penicillin G, 50 μg/ml of streptomycin (Sigma) and 2% heat-inactivated horse serum (Sigma) at 37 °C in a 5% CO_2_ humidified incubator. Tachyzoites were washed in phosphate-buffered saline (PBS; pH 7.4) and stored at −70 °C for the subsequent extraction of DNA.

### 2.2 Genomic DNA preparation

Tachyzoites of *N. caninum* were lysed in 2 ml of lysis buffer containing 100 mM of EDTA, 10 mM of Tris–Cl (pH 7.6), 1% of SDS containing 40 U of proteinase K [50 mg/ml] (Sigma) at 65 °C for 4 h. Genomic DNA was purified by extraction with phenol–chloroform, followed by ethanol precipitation. The DNA obtained was quantified using a Nanodrop spectrophotometer and stored at −20 °C until used in PCR.

### 2.3 Identification of mini and microsatellite sequences in *N. caninum* DNA

Approximately 25,000 expressed sequence tags (ESTs) of *N. caninum* were screened for repetitive sequences using the program ETANDEM (http://bioweb.pasteur.fr/seqanal/interfaces/etandem.html) (Table 2). Primer pairs were designed (that flank and amplify a sequence containing the repeat) for 27 of the tandemly repeated sequences identified using Primer 3 [20] (http://frodo.wi.mit.edu/cgi-bin/primer3/primer3_www.cgi).

### 2.4 PCR of repetitive DNA and DNA sequencing

Standard PCR reagents (Fisher Biotec, Australia) were used. The PCR reaction mixture (50 μl) contained 1× DNA polymerase reaction buffer, 0.16 mM of dNTPs, different amounts of MgCl_2_ (Table 2), 1 U of *Taq* DNA polymerase and 0.5 μM of each primer (Sigma Genosys). A number of controls were included in each run, including a negative control (designated “ddH_2_O blank”), and reactions containing either Vero or *Toxoplasma gondii* DNA.

PCR was performed in a PTC-100 thermocycler (MJ Research Inc.) using the following conditions: 95 °C for 5 min; 25 cycles of 94 °C for 1 min; different annealing temperatures (Table 2) were applied for 1 min; 72 °C for 1 min followed by 1 cycle of 72 °C for 5 min. PCR products were separated electrophoretically on 1.5% agarose gels using pGEM (Promega) markers. Products were then stained using ethidium bromide and detected by UV transillumination.

A gel purification kit (QIAquick Gel Extraction, Qiagen) was used to purify PCR products by following the manufacturer's instructions. DNA sequencing, using both forward and reverse primers, was performed by SUPAMAC (University of Sydney, Australia). From these sequences, a consensus sequence was obtained for each locus for each isolate. The program ClustalW (http://www.ebi.ac.uk/Tools/clustalw/index.html) was then used to align the sequences from all isolates studied, in order to detect any nucleotide variation that might exist amongst them.

### 2.5 Multiplex PCR typing of *N. caninum* isolates

Three different primer pairs were included in the multiplex PCR. Primer pairs to DNA regions Tand-12 and Tand-13 were identified in our study here and a primer pair to region Tand-3 was reported previously by Regidor-Cerrillo et al. [12].

For the multiplex PCR reaction, the same conditions were used as described above, except that the three pairs of primers (0.5 μM for each primer) were included together, along with 1.5 mM of MgCl_2_ and 1.5 U of *Taq* DNA polymerase. The PCR was conducted using the annealing temperature of 61 °C for 1 min, for 25 cycles.

Twenty-five isolates of *N. caninum* from different geographical regions of the world were subjected to analysis (see Table 1) as well as *T. gondii* (ME49 isolate) and 12 isolates of *Hammondia heydorni* (kindly provided by Dr G. Schares; HY Giessen-1999, HY2, HY3, HY4, HY5, HY6, HY7, HY8, HY9, HY10, HY11, HY12).

Analytical sensitivity of the PCR was investigated. A series of 10-fold titrations of tachyzoite DNA were prepared ranging from 100 ng to 0.0001 ng per reaction. DNA from one tachyzoite equates to ∼0.0001 ng [21].

To determine the effect of parasite culture on the multiplex PCR, an aliquot of tachyzoites from a seed stock of NC-Nowra maintained in liquid nitrogen was thawed and seeded into culture (Vero cells). DNA was then extracted from the cultured tachyzoites at various time points over a 12-month period and analysed by multiplex PCR. The PCR products obtained were analysed by agarose gel electrophoresis.

### 2.6 Transplacental transmission of *N. caninum*

Previous studies using an immunisation/challenge mouse model [22], demonstrated that infections of NC-Nowra that established before pregnancy effectively prevented transplacental transmission of a challenge infection by NC-Liverpool given during pregnancy. However, in this study [22], *N. caninum* DNA was detected in the brain of a small number of pups. It was unknown whether the strain used to immunize or challenge was transmitted to the foetus during pregnancy. Therefore, multiplex PCR was used to determine the identity of the parasite transmitted in those experiments from the DNA extracted from the brains of the pups. The PCR products obtained were analysed by agarose gel electrophoresis.

### 2.7 Characterisation of *N. caninum* DNA from sera from infected animals

Sera from four dogs from a dairy farm in New South Wales, Australia, were collected previously and stored at −20 °C. These sera were examined for *N. caninum* antibodies by indirect immunofluorescent antibody test (IFAT) [23]. One female cattle dog (18 months of age) had an IFAT titre of 1:200 against *N. caninum*. She subsequently gave birth to six pups and the bitch was bled for serological and DNA testing for *N. caninum*. Serum was prepared by allowing blood to clot overnight, and DNA extracted from the serum according to the same method as described in Section 2.2. Multiplex PCR (using primer pairs to satellites Tand-3, Tand-12 and Tand-13) was performed on the DNA extracted, and the PCR products were detected by gel electrophoresis. This multiplex PCR was run for 35 cycles, instead of 25 cycles.

## 3 Results

### 3.1 Characterization of repetitive sequences

Twenty-seven primer pairs were designed from the EST sequence data to PCR sequences from the *N. caninum* genome (Table 2). Sixteen primer pairs produced single PCR products, and no variation was detected amongst *N. caninum* strains using eight of these targets. Other primers amplified multiple bands or did not yield products (see Table 2). Eight primer pairs amplified PCR products which were polymorphic in size, assessed initially using DNA from NC-Liverpool, NC-Nowra, NC-Illinois, NC-SweB1, BPA1, NC-Beef, JAP1 and NC1 isolates. Sequencing determined the nature and extent of the diversity detected in DNA regions Tand-4, Tand-8, Tand-12, Tand-13, Tand-15, Tand-16, Tand-30 and Tand-32 (see Table 3). The nucleotide sequences of the repetitive DNAs have been deposited in the GenBank database under accession numbers: Tand-3, FJ824912–FJ824936FJ824912FJ824913FJ824914FJ824915FJ824916FJ824917FJ824918FJ824919FJ824920FJ824921FJ824922FJ824923FJ824924FJ824925FJ824926FJ824927FJ824928FJ824929FJ824930FJ824931FJ824932FJ824933FJ824934FJ824935FJ824936; Tand-4, FJ824993–FJ824998FJ824993FJ824994FJ824995FJ824996FJ824997FJ824998; Tand-8, FJ824937–FJ824942FJ824937FJ824938FJ824939FJ824940FJ824941FJ824942; Tand-9, FJ830453–FJ830458FJ830453FJ830454FJ830455FJ830456FJ830457FJ830458; Tand-12, FJ824943–FJ824967FJ824943FJ824944FJ824945FJ824946FJ824947FJ824948FJ824949FJ824950FJ824951FJ824952FJ824953FJ824954FJ824955FJ824956FJ824957FJ824958FJ824959FJ824960FJ824961FJ824962FJ824963FJ824964FJ824965FJ824966FJ824967; Tand-13, FJ824968–FJ824992FJ824968FJ824969FJ824970FJ824971FJ824972FJ824973FJ824974FJ824975FJ824976FJ824977FJ824978FJ824979FJ824980FJ824981FJ824982FJ824983FJ824984FJ824985FJ824986FJ824987FJ824988FJ824989FJ824990FJ824991FJ824992; Tand-15, FJ824999–FJ825003FJ824999FJ825000FJ825001FJ825002FJ825003; Tand-16, FJ825004–FJ825007FJ825004FJ825005FJ825006FJ825007; Tand-23, FJ825008–FJ825012FJ825008FJ825009FJ825010FJ825011FJ825012; Tand-24, FJ830459–FJ830464FJ830459FJ830460FJ830461FJ830462FJ830463FJ830464; Tand-25, FJ830465–FJ830468FJ830465FJ830466FJ830467FJ830468; Tand-26, FJ830469–FJ830473FJ830469FJ830470FJ830471FJ830472FJ830473; Tand-30, FJ830474–FJ830480FJ830474FJ830475FJ830476FJ830477FJ830478FJ830479FJ830480; Tand-31, FJ830481–FJ830487FJ830481FJ830482FJ830483FJ830484FJ830485FJ830486FJ830487; Tand-32, FJ825013–FJ825017FJ825013FJ825014FJ825015FJ825016FJ825017; Tand-34, FJ830488–FJ830489FJ830488FJ830489.

Region Tand-4 had a sequence containing a 33 bp tandemly repeated minisatellite; Tand-12 had a sequence containing a 25 bp minisatellite repeat and Tand-13 contained a 21 bp minisatellite repeat. Tand-8, Tand-15, and Tand-16 yielded a sequence containing a microsatellite of (AT)*n* bp. Tand-30 had a sequence containing a microsatellite of (TATC)*n*(TA)*n*, whereas Tand-32 possessed a sequence containing a microsatellite of (TA)*n*(GA)*n*. Using these primer pairs, no PCR product was obtained from DNA of *T. gondii* or Vero cells.

**Fig. 1:**
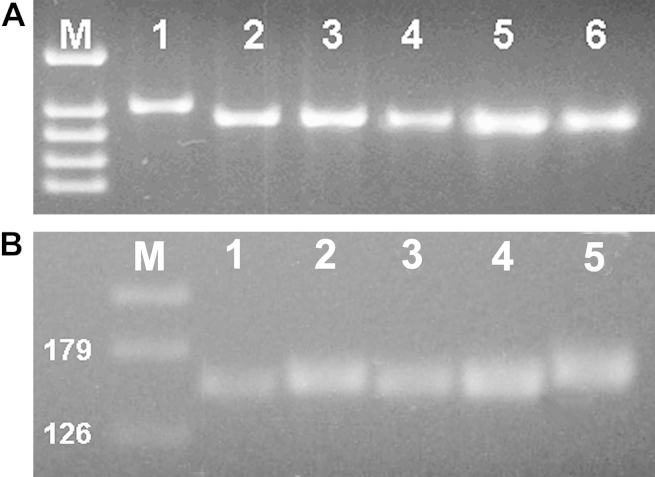
PCR amplification of (A) Tand-4 and (B) Tand-15. (A) Lanes: M = marker, 1 = NC1, 2 = NC-Beef, 3 = BPA1, 4 = NC-SweB1, 5 = NC-Nowra, 6 = NC-Liverpool. (B) Lanes: M = marker, 1 = BPA1, 2 = NC-SweB1, 3 = NC-Illinois, 4 = NC-Nowra, 5 = NC-Liverpool. The pictures show the outcome of a preliminary screen for diversity amongst isolates in their repetitive DNA.

Analyses of satellite region Tand-4 demonstrated that NC1 gave a unique profile; all other isolates studied had four copies of the repeat, while NC1 possessed five copies (see Table 3 and Fig. 1A). Similar analyses of Tand-15 showed that NC-Nowra and NC-Illinois contained a similar number of repeats at this locus (see Table 3 and Fig. 1B). More diversity was detected in Tand-12 and Tand-13, which were studied in more detail.

**Fig. 2:**
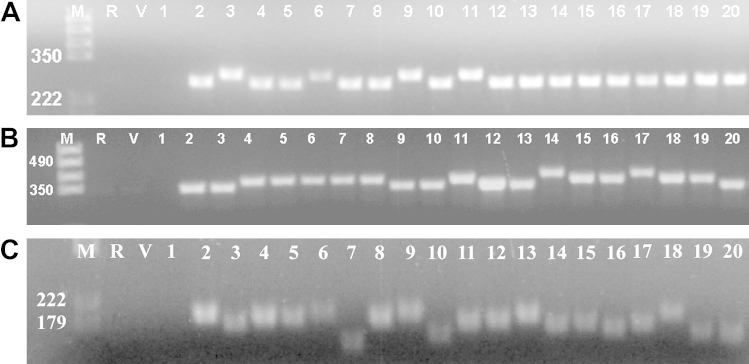
PCR amplification of repetitive sequences from different *N. caninum* isolates. (A) Tand-12; (B) Tand-13; (C) Tand-3. Lanes M = marker (given in bp), R = control, V = Vero, 1 = *T. gondii*, 2 = NC-Liverpool, 3 = NC-Nowra, 4 = NC-SweB1, 5 = BPA1, 6 = BPA6, 7 = NC-Beef, 8 = JAP1, 9 = WA-K9, 10 = NC-Bahia, 11 = NC-LivB1, 12 = NC-LivB2, 13 = NC-GER1, 14 = NC-GER2, 15 = NC-GER3, 16 = NC-GER4, 17 = NC-GER5, 18 = NC-GER6, 19 = NC-GER8, 20 = NC-GER9.

**Fig. 3:**
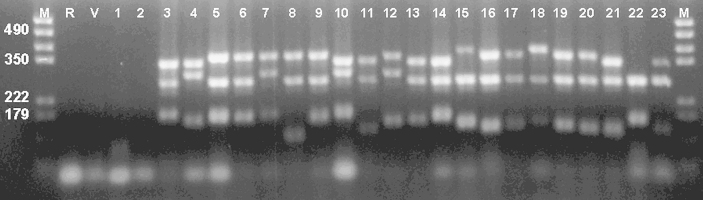
Multiplex PCR was performed for the three markers: Tand-3 Tand-12 and Tand-13. Lanes: M = marker (given in bp), R = control, V = Vero, 1 = *T. gondii*, 2 = *Hammondia heydorni*, 3 = NC-Liverpool, 4 = NC-Nowra, 5 = NC-SweB1, 6 = BPA1, 7 = BPA6, 8 = NC-Beef, 9 = JAP1, 10 = WA-K9, 11 = NC-Bahia, 12 = NC-LivB1, 13 = NC-LivB2, 14 = NC-GER1, 15 = NC-GER2, 16 = NC-GER3, 17 = NC-GER4, 18 = NC-GER5, 19 = NC-GER6, 20 = NC-GER8, 21 = NC-GER9, 22 = NC-Illinois, 23 = NC1.

The isolates were divided into two groups based on sequence data for the Tand-12 region (Table 3; Figs. 2A and 3); a group with three repeats and the other with four repeats. All eight isolates of this parasite from Germany (NC-GER1 to NC-GER9) possessed the same number of repeated sequences (*n* = 3).

Isolates of *N. caninum* were divided into three groups using sequence data for the Tand-13 region (Table 3; Figs. 2B and 3); the first group with four repeat copies, the second group with five and the last one with six copies.

According to Regidor-Cerrillo et al. [12], region Tand-3 exhibits high polymorphism of three different tandemly repeated microsatellites [(ACT)*n* (AGA)*n* (TGA)*n*]. We verified this observation during this study. Tand-3 sequences exhibited a high level of polymorphism (Table 3; Figs. 2C and 3), with many of the isolates showing a unique pattern of polymorphism. The following groups of isolates, however, shared the same sequences in region Tand-3: NC-GER4 and NC-GER6, NC1 and NcIs491, the New Zealand isolates (NcNZ1, NcNZ2 and NcNZ3) and NC-Liverpool.

### 3.2 Use of multiplex PCR for the characterization of *N. caninum* isolates

For multiplex PCR, three pairs of primers were used in a single PCR reaction; two targeting minisatellite (Tand-12 and Tand-13) and one microsatellite (Tand-3) described by Regidor-Cerrillo et al. [12]. Twenty-five isolates of *N. caninum* were studied by multiplex PCR and different levels of polymorphism were detectable. Using the multiplex PCR, no product was detected using Vero, *T. gondii* (ME49 isolates) or *H. heydorni* DNA (Fig. 3). Some of the isolates gave identical DNA profiles indicative of similar number of repeats present at the loci studied (Table 3 and Fig. 3). NC-Illinois PCR produced two visible bands which belong to Tand-3 and Tand-12 while Tand-13 was poorly amplified.

**Fig. 4:**
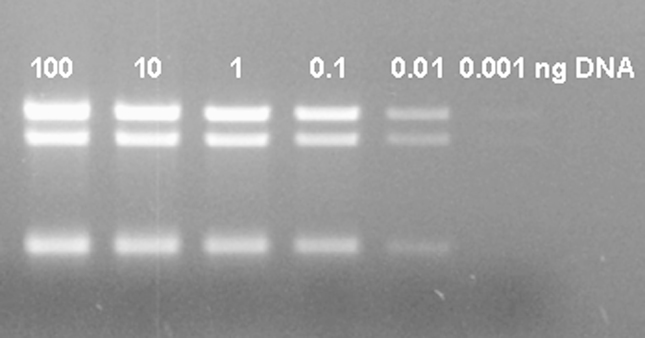
Sensitivity of multiplex PCR analysis assessed using different amounts of purified NC-Nowra DNA as template.

Applications of the multiplex PCR were investigated. In an assay for PCR sensitivity, 10 pg of parasite DNA was detected using serial titrations of purified DNA (Fig. 4). This amount of parasite DNA equals ∼100 tachyzoites.

No differences in DNA profiles were detected by multiplex PCR (not shown) between DNA of NC-Nowra extracted from in vitro cultures from our laboratory over a one-year period. These results suggest that a multiplex PCR profile is a stable trait for cultured tachyzoites.

**Fig. 5:**
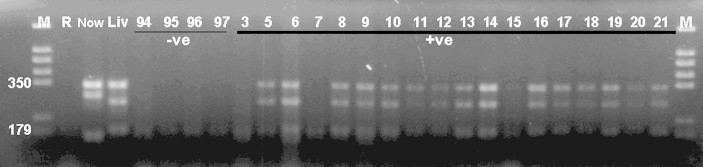
Multiplex PCR of DNA extracted from pup brains showing that NC-Liverpool isolate is present in the brains. Lanes: M = marker (given in bp), R = control, Now = NC-Nowra, Liv = NC-Liverpool, 94–97 = negative control of mouse brain, 3–21 = mouse brains from a challenge experiment infected with NC-Liverpool.

Multiplex PCR of DNA extracted from mice pup brains, from the study by Miller et al. [22], showed that NC-Liverpool DNA was present in brains previously identified to contain *N. caninum* DNA (see Fig. 5); DNA from NC-Nowra was not detected. No PCR products were generated from four DNAs derived from pup brains, which were originally classified as test-negative for *N. caninum* DNA. Hence, we conclude that the NC-Liverpool challenge strain was transmitted to the pups during the vaccine experiments [22].

**Fig. 6:**
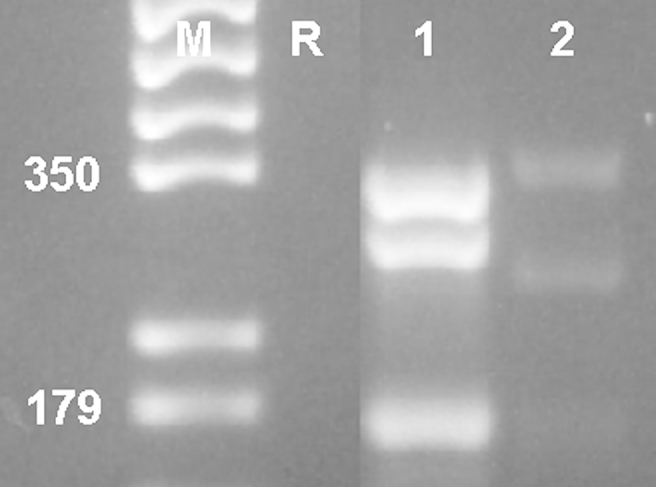
Multiplex PCR of DNA extracted from dog serum that was antibody positive to *N. caninum*. Lanes: M = marker (given in bp), 1 = DNA from NC-Nowra, 2 = DNA from dog serum.

Analyses of genomic DNA isolated from serum from an *N. caninum*-infected dog showed that the DNA can be amplified by multiplex PCR (Fig. 6), and that specimens can be readily compared. The sequence obtained from the PCR products from the serum DNA was identical in sequence to those obtained from the NC1 and NcIs491 isolates. Therefore, the multiplex technology described here is considered a suitable tool for the detection of animals infected with *N. caninum* as well as for future studies of genetic diversity within this species.

## 4 Discussion

Regidor-Cerrillo et al. [12] reported differences in 12 microsatellite markers [predominantly (AT)*n*] among nine isolates of *N. caninum*. Multilocus analysis showed that each of the nine isolates displayed a distinctive profile, but revealed no relationship between genotype and host or geographical origin. The goal of this study was to describe the presence of mini and microsatellite DNAs in the genome of *N. caninum* and to investigate genetic diversity associated with them. Extensive genetic diversity was detected within the species *N. caninum*. Sixteen sequences containing repeats were studied in detail and polymorphism was detected. The number of repeat elements present varied amongst isolates (see Table 3).

A key outcome from this study was the development of a multiplex PCR that has a practical use for the detection of genetic variants of *N. caninum*. Primers for two minisatellites, defined in the present study, and a third, highly variable microsatellite target (Tand-3) selected from a previous study [12] were included in the assay. The assay is specific and sensitive, and the results obtained were stable when cultured organisms were compared. We also showed that most isolates (except NC-GER4 and NC-GER6, NC1 and NcIs491, NC-Liverpool and the New Zealand isolates NcNZ1, NcNZ2, NcNZ3) have their own unique PCR profile at Tand-3. The German isolates, NC-GER4 and NC-GER6, are also similar to HY-Berlin-1996 (*N. caninum*) at Tand-3 [12], which suggests this variant may be more common in nature. Further studies are needed in this area to investigate diversity of field isolates.

The microsatellite Tand-3 was first described by Regidor-Cerrillo et al. [12] as a marker called MS10. Subsequently it was used to genotype a range of isolates [12,35,40]. However, the present results differ slightly from those reported for two of the isolates studied. For NC-SweB1 and NC-GER1, the profile obtained by Regidor-Cerrillo et al. [12] was [(ACT)_6_ (AGA)_14_ (TGA)_9_] and [(ACT)_6_ (AGA)_25_ (TGA)_9_], respectively, which is different from the results obtained in the current study [(ACT)_8_ (AGA)_23_ (TGA)_8_] and [(ACT)_6_ (AGA)_24_ (TGA)_9_], respectively. For NC-GER9, the profile obtained from our study [(ACT)_6_ (AGA)_12_ (TGA)_9_] was different from that reported by Basso et al. [35] which was [(ACT)_6_ (AGA)_22_ (TGA)_8_]. Also, the repeat profile for isolate NC-PV1 [12] was identical to JAP1 (present study), NC-Spain H1 [40], NC-Spain9 and NC-Spain10 [41]. The profile of BPA1 (present study) was identical to KBA2 [12] and NC-Spain7 [41]. The German isolates NC-GER3 and NC-GER5 (present study) were identical to NC-Spain 5H and NC-Spain8 [41], respectively. Some similarities in the repeat content of different isolates are starting to emerge from such molecular studies.

RAPD-PCR was used to study the diversity of parasite populations including *N. caninum* [7,8,29,42]. Although RAPD-PCR analysis may have the ability to differentiate between individual isolates, this technique suffers from a lack of specificity due to use of low annealing temperatures and the size of primers [12].

Few differences were reported in the first internal transcribed spacer (ITS-1) region among different isolates of *N. caninum*. Intra-strain differences were reported for the NC-Bahia strain ITS-1 sequence analysis which differs in 12 bp from those of North American and European strains [11], while no major differences were identified in sequence among 11 other *N. caninum* isolates [10,19,21,43–47]. Thus, the ITS-1 region is not sufficiently variable in sequence for studying diversity within the genus *Neospora* [11]. This statement also applies to regions in the alpha-tubulin, beta-tubulin and heat shock protein 70 (HSP-70) genes, with no variation detected among different isolates of *N. caninum* [37,48].

A multiplex PCR assay was developed previously [49] for the *ante mortem* diagnosis of toxoplasmosis and neosporosis in samples from dogs and cats. They amplified the canine ferritin gene and feline histone 3.3 gene in a single PCR. A multiplex assay, using five primer pairs, was also developed for the characterization of strains of *T. gondii* [14] and applied to the genetic analysis of *Toxoplasma* isolates from humans and other animals [50]. A nested multiplex PCR-RFLP was developed [51] using four independent polymorphic markers and applied to detect genetic diversity in *T. gondii* from immunocompromised patients and human congenital toxoplasmosis [52]. No previous studies describe the development or application of multiplex PCR for study of genetic diversity amongst *N. caninum* isolates.

An interesting application of the present multiplex PCR is its use for detecting the presence of isolate-specific DNA profiles linked to biological specimens. For samples with mixed infections of different isolates of this parasite, this might have limitations. The result of such an assay depends on which isolate's DNA is the most abundant in the sample. We applied the multiplex PCR to the detection of *N. caninum* DNA in the brains of mice pups produced from an immunisation/challenge experiment [22]. In this experiment, mice were vaccinated before pregnancy with an infection of NC-Nowra and then challenged during pregnancy with an infection of NC-Liverpool. A small number of pups contained genomic DNA of *N. caninum*; here, we demonstrated that this DNA was derived from the challenge strain NC-Liverpool.

The present multiplex PCR might also have future utility as a diagnostic tool, given that *N. caninum* DNA in animal serum could be detected and characterized. Hence, this technology potentially has a wide range of applications. One additional possible use might be in studying animals vaccinated with a live *N. caninum* vaccine [53] and where a “vaccine break-down” or “reversion to virulence” of the vaccine strain is suspected. The multiplex PCR may be able to attribute abortions or infections to a particular strain.

In conclusion, a multiplex PCR assay was developed that is able to identify genetic diversity among *N. caninum* isolates. This method is simple, as only a single PCR is performed for three different loci. This method is rapid and the typing procedure can be performed within one day. The PCR is highly informative and sensitive, and can readily distinguish amongst isolates.

## Figures and Tables

**Table 1:** Isolates of *Neospora caninum* used in this study.

Isolate	Host	Country	Reference
NC-1	Brain of congenitally infected dog	United States	[1]
BPA1	Brain of aborted bovine foetuses	United States	[24]
BPA6	Brain and/or spinal cord of an aborted bovine foetus	United States	[25]
NC-Beef	Naturally infected calf	United States	[26]
NC-Illinois	Brain of infected diary calf	United States	[27]
NC-Liverpool	Cerebrum of congenitally infected dog's pup	United Kingdom	[19,28]
NC-LivB1	Brain of stillborn calf	United Kingdom	[29]
NC-LivB2	Brain of aborted bovine foetus	United Kingdom	[30]
NC-SweB1	Brain of stillborn calf	Sweden	[31]
JAP1	Brain and spinal cord of congenitally infected calf	Japan	[32]
NC-GER1	Brain and spinal cord of congenitally infected dog's pup	Germany	[33]
NC-GER2, 3, 4, 5 and NC-GER6	Oocysts from naturally infected dog	Germany	[34]
NC-GER8 and NC-GER9	Oocysts from naturally infected dog	Germany	[35]
NC-Bahia	Brain of naturally infected adult dog	Brazil	[36]
NC-Nowra	Brain and spinal cord of congenitally infected calf	Australia	[10]
WA-K9	Skin lesions of naturally infected dog	Australia	[37]
NcNZ1	Brain of naturally infected cow	New Zealand	[38]
NcNZ2	Brain of two days old calf		
NcNZ3	Brain of stillborn calf		
NcIs491	Brain of aborted bovine foetus	Israel	[39]

**Table 2:** A summary of the mini and microsatellite loci studied and their PCR amplification conditions.

Locus	Accession no.	Source^a^	Repeat copy number	Size of repeat (bp)/expected size of PCR product	PCR primers (5′–3′)^b^	Temp^c^ (°C)	Vol. of MgCl_2_ 2 mM (μl)d,e	Type of band^e^
Tand-3	CF775292	NC1	(ACT)_**7**_ (AGA)_**12**_ (TGA)_**9**_	3/140	**F**: CCCTCGTGTCGTACTCGTAG	61	3	S
					**R**: CCCTGTTTGACGTAGATTGA			
Tand-4	CF940315	NC1	(CCTCGTCTCCTGAGCCCTCAGGTTGTGGTCGAA)_**5**_	33/525	**F**: GTTTTCACGTTATCAGGCCG	63	5	S
					**R**: GTGCTCGATGTGCCGGCG			
Tand-5	CF940908	NC1	(TCACTGTGTGGGCGAATGCTGTCTCAGACTTCCAG)_**4**_	35/554	**F**: AATTCTACGGCAATCGCGG	60	5	D
					**R**: ACTGGAAGTCTGAGACAGCA			
Tand-7	BF249062	NC1	(AG)_**29**_	2/616	**F**: GAGAGAGAGAGAGAGAGAGAGA	55	T	N
					**R**: CCAATCCGGTAGTAAGACAT			
Tand-8	CF967185	NC1	(AT)_**25**_	2/301	**F**: CATGCCAAGAATTCGACAGA	61	3	S
					**R**: ACGCATCGGAGAAGAGAGAA			
Tand-9	CF659795	NC1	(CAGGAGTCTCTGCTACCGAAGAGACAT)_**3**_	27/230	**F**: CAGGACACAGGGGAAACAGT	61	3	S
					**R**: ACCCCTTATTCAGCGTTGC			
Tand-10	CF274074	NC-Liv	(GAGGCTTCTCCCGACACCGCGTCGACCGAGGGC)_**3**_	33/490	**F**: GCTTCTCCCGACACCGCGTC	57	5	M
					**R**: CTGCGCTCGGAGGTCTTTGT			
Tand-11	CF598557	NC-Liv	(GGCGTGGCTTCTCCCGACACCGCGTCGACCGAG)_**3**_**and** (CGCGTCGACCGAGGGCGTGGCTTCTCCCGACAC)_**3**_	33/159	**F**: GACCGAGGGCGAGGCTTCT	61	6	D
					**R**: CCGCCGTCAGGATAAAACACA			
Tand-12	CF939461	NC1	(AGTTTTGCCGTTTTGCTAACGTGAA)_**3**_	25/277	**F**: CCCGCATTACCCTTGTTG	61	3	S
					**R**: CTAGGATGCACACGGACACA			
Tand-13	CF260222	NC-Liv	(CGTCGCCTCCCGCCGACAGTG)_4_	21/340	**F**: GGCTGATCCGCTCTGTGAAA	61	3	S
					**R**: TTCCCCCCTCGCAAAGTC			
Tand-14	CF775542	NC1	(TA)_**16**_ TG (TA)_**4**_ TG (TA)_**2**_ (TG)_**2**_ (TA)_**2**_ [TG (TA)_**3**_]_**3**_	2/432	**F**: CAGAAGATGACCAGAGCGAT	55	5	M
					**R**: CCATATATACATATATACATATATACA			
Tand-15	CF599297	NC-Liv	(TA)_**8**_ TG (TA)_**13**_	2/159	**F**: GCTTTCCCGGCATTTGTTCG	61	3	S
					**R**: CACTTTGACCTACACAGATACACA			
Tand-16	CF260759	NC-Liv	(AT)_**9**_ GT (AT)_**9**_ (AT)_5_ (ACAT)_3_ GCA (GT)_5_	2 and 4/176	**F**: CGACTGCCAGCTCCGGAAGG	61	3	S
					**R**: GAGCCAGGCACGGAGGTAGA			
Tand-19	CF968042	NC1	(AT)_**5**_ GT (AT)_**11**_ CT GT (AT)_**2**_ (GT)_**3**_ AT GT AT GC (AT)_**9**_	2/170	**F**: GTCCTACCGTCCGTTTCCTC	61	T	N
					**R**: CGCCTATCCCTCAGCATAAA			
Tand-21	CF422598	NC-Liv	(GTCGTCGGAGCAAGACTCGAAGGGAGCGGAAAC)_**3**_	33/197	**F**: AAAAGTCGTCGGAGCAAGG	59	4	M
					**R**: GACGACGTTTCTGCTCCATT			
Tand-22	CF422423	NC-Liv	(TCCGCCGGTTCCTCCGCGTGACCCAAT)_**3**_	27/217	**F**: CTGGAAGGAAAGGGAAGGAC	61	5	S
					**R**: ATTGGATCACGCGGAGGAAC			
Tand-23	CF261117	NC-Liv	(GAAGAAGGGAGACGTGCGAGGAATTAGACGGGAAGAACTGA)_**3**_	41/608	**F**: AGATCTATCGCCGCACCTCG	61	3	S
					**R**: TTCTGTCCGCGTCGTCTTTC			
Tand-24	CF798073	NC1	(GGTCGAGGAGGC)_**4**_	12/200	**F**: GATGGCAGGACCGTGTGGAT	61	3	S
					**R**: TCTTTGGCCGCCTTCACG			
Tand-25	CF939363	NC1	(AACAGTCTGCCCCGTACA)_**3**_	18/158	**F**: TGCCTTTCTCTCGCGCTTCT	61	3	S
					**R**: TGTGCAAGGGGATTGGAGTG			
Tand-26	CF598061	NC-Liv	(CTCTCTCCGCTCGTCGTCTG)_**3**_**and** (CGTCGTCTGCTCTCTCCGCT)_**4**_	20/186	**F**: TGGTTGTTCTGCCGGTATCTCC	61	3	S
					**R**: GGGAACGGAAGAGAGGAGACG			
Tand-28	CA857134	NC1	(GTAAGAATAGGTATCAAGT)_**3**_	19/230	**F**: GTAAGAATAGGTATCAAGTGTAAGAA	55	T	N
					**R**: GGAACGGACTCAACTGTGTA			
Tand-29	CF273699	NC-Liv	(AGCGCCAGCGGAGAGT)_**3**_	16/150	**F**: AGATGGAGAAGTCGCGAGAG	61	3	M
					**R**: ATGCTACCGATCTCCTCACC			
Tand-30	BF248567	NC1	(TATC)_**5**_ (TA)_**12**_	4 and 2/444	**F**: TTCCAAAACAGCGACCCACT	59	5	S
					**R**: GTTCACGACGGTAATCCTTT			
Tand-31	CF659048	NC1	(CCT)_**2**_ CTT CCT [CTT (CCT)_**2**_]_**3**_ CTT CCT CTT (CCT)_**2**_ (CTT)_**2**_ CCT CTT (CCT)_**2**_ (CTT)_**3**_ [CCT (CTT)_**5**_]_**2**_ [CCT (CTT)_**4**_]_**2**_ CCG (CTT)_**3**_ (CCT)_**2**_	3/444	**F**: TCCCACGACACCCAACACCT	61	3	S
					**R**: GAGGAGGAAGAAGAAGCGGAAGA			
Tand-32	CF598773	NC-Liv	(TA)_**8**_ (GA)_**10**_	2/150	**F**: GCATTTCACGCATCACCTAACA	61	3	S
					**R**: CATGTACGCTCAAATCTCTCTCTCTC			
Tand-34	CF796838	NC1	(CTC)_**3**_ CCT (CTT)_**2**_ (CCT)_**4**_ CTT (CCT)_**3**_ CTT (CCT)_**2**_ CTT CCT CTT (CCT)_**2**_ CTT (CCT)_**2**_ (CTT)_**2**_	3/131	**F**: CGCTCTACCCCTCCTCCTC	61	3	S
					**R**: CGCTCCCTGTCTCGTGATT			
Tand-35	CF260238	NC-Liv	(TC)_**10**_ (AT)_**11**_	2/210	**F**: GTTCCGTCTTCTGCGAGGT	61	3	M
					**R**: GGAAGAGGCTCAGATGCAAA			

a Isolate of *N. caninum* from which the sequence data were derived.

b F, forward primer; R, reverse primer.

c PCR annealing temperature.

d Volume added to the PCR reaction for optimal product production.

e S, single band; D, double band; N, no band; M, multiple band; T, all MgCl_2_ titrations.

**Table 3:** Repetitive sequences found in different *N. caninum* isolates following sequencing of PCR products.

Isolate	Copy number of repeats								
	Tand-4	Tand-3	Tand-8	Tand-12	Tand-13	Tand-15	Tand-16	Tand-30	Tand-32
NC-Liverpool	4	(ACT)_6_ (AGA)_26_ (TGA)_10_	(AT)_12_	3	4	(TA)_8_TG(TA)_13_	(AT)_9_ GT (AT)_9_	(TATC)_7_ (TA)_10_	(TA)_8_ (GA)_10_
NC-Nowra	4	(ACT)_6_ (AGA)_22_ (TGA)_8_	(AT)_8_	4	4	(TA)_8_TG(TA)_10_	(AT)_9_ GT (AT)_9_	(TATC)_5_ (TA)_12_	(TA)_10_ (GA)_8_
NC-Illinois		(ACT)_6_ (AGA)_20_ (TGA)_10_	(AT)_9_	3	4	(TA)_8_TG(TA)_10_	(AT)_8_ GT (AT)_11_	(TATC)_6_ (TA)_14_	
NC-SweB1	4	(ACT)_8_ (AGA)_23_ (TGA)_8_		3	5	(TA)_9_TG(TA)_10_	(AT)_9_ GT (AT)_9_	(TATC)_6_ (TA)_11_	(TA)_9_ (GA)_9_
BPA1		(ACT)_6_ (AGA)_23_ (TGA)_10_	(AT)_12_	3	5	(TA)_8_TG(TA)_9_	(AT)_8_ GT (AT)_9_	(TATC)_6_ (TA)_17_	(TA)_10_ (GA)_10_
BPA6		(ACT)_6_ (AGA)_26_ (TGA)_9_		4	5				
NC-Beef	4	(ACT)_6_ (AGA)_11_ (TGA)_7_	(AT)_11_	3	5				(TA)_9_ (GA)_10_
NC1	5	(ACT)_7_ (AGA)_12_ (TGA)_9_		3	4			(TATC)_5_ (TA)_12_	
JAP1		(ACT)_6_ (AGA)_21_ (TGA)_10_	(AT)_12_	3	5		(AT)_8_ GT (AT)_9_	(TATC)_6_ (TA)_17_	(TA)_10_ (GA)_10_
WA-K9		(ACT)_6_ (AGA)_26_ (TGA)_8_		4	4				
NC-Bahia		(ACT)_5_ (AGA)_14_ (TGA)_9_		3	4				
Nc-LivB1		(ACT)_6_ (AGA)_21_ (TGA)_8_		4	5				
Nc-LivB2		(ACT)_7_ (AGA)_20_ (TGA)_9_		3	4				
NC-GER		(ACT)_6_ (AGA)_24_ (TGA)_9_		3	4				
NC-GER		(ACT)_6_ (AGA)_18_ (TGA)_9_		3	6				
NC-GER		(ACT)_6_ (AGA)_16_ (TGA)_8_		3	5				
NC-GER		(ACT)_6_ (AGA)_17_ (TGA)_8_		3	5				
NC-GER		(ACT)_6_ (AGA)_18_ (TGA)_10_		3	6				
NC-GER		(ACT)_6_ (AGA)_17_ (TGA)_8_		3	5				
NC-GER		(ACT)_6_ (AGA)_15_ (TGA)_8_		3	5				
NC-GER		(ACT)_6_ (AGA)_12_ (TGA)_9_		3	4				
NcNZ1		(ACT)_6_ (AGA)_26_ (TGA)_10_		3	4				
NcNZ2		(ACT)_6_ (AGA)_26_ (TGA)_10_		3	4				
NcNZ3		(ACT)_6_ (AGA)_26_ (TGA)_10_		3	4				
NcIs491		(ACT)_7_ (AGA)_12_ (TGA)_9_		3	4				
